# Choledochoduodenostomy in the Management of Common Duct Stones or Associated Pathology –  An Obsolete Method?

**DOI:** 10.1155/1996/47821

**Published:** 1996

**Authors:** António Castro Mendes de Almeida, Noel Medina dos Santos, Fernando José Aldeia

**Affiliations:** University Hospital of Santa Maria – Medicina Operatória Lisbon Medical School Av.Egas Moniz Lisboa Codex 1699 Portugal

## Abstract

Choledochoduodenostomy (CDD) has been reported as a more effective treatment of CBD stones than T-tube drainage but it is regarded as a last resort or obsolete therapeutic method due to fears of higher mobidity, cholangitis, “sump” syndrome and liver dysfunction. We aimed to assess the aforementioned issues analyzing prospectively our experience from 1976 through Dec.92.

*Methods*: CDD was performed in 89 females and 36 males, aged 60.2±8.7 years, 26 during repeat surgery. Duct stones were the indication in 94, Sphincter of oddi (SO) dysfunction in 23 and obstructive pancreatitis nodule in 8. Peroperative liver biopsies were obtained in 44 patients. The “follow-up” schedule (> 2.5 years in 110) included clinical interview and LFT's on an yearly basis. Ultra sound (USG) was obtained every one or two years. ERC was done in 10 symptomatic patients and in 25 others for protocul purposes. Liver biopsies were taken four to nine years post surgery in 11 patients-five at relaparotomy for non-biliary causes and six percutaneously by fine needle. Ductal mucosa biopsy could safely be performed in one patient 10 years after surgery. The long-term results were classified as *excellent, good, fair* or *poor*. *Poor* meant the need for further invasive therapy (resurgery or EST).

*Results*: There were two operative deaths (1.6%). The long-term results (123 survivors) were considered *excellent* in 89, *good* in 22, *fair* in 9 and *poor* in three. Three patients died from unrelated causes and eight others ceased the “follow-up” evaluation three to five years post surgery. All of them were considered as having *excellent* or *good* results. A widely patent anastomosis of approximately 20 mms without mucosal inflammatory changes was documented in every patient assessed via ERC. food “debris” was detected within the distal duct of four patients yet it was easily flushed through the stoma. Normal tissue patterns were observed in all long-term liver biopsies. Likewise the ductal mucosa biopsy failed to reveal any acute or chronic inflammatory changes.

*Conclusions:* 1) CDD is ahighly effective short and long-term treatment of CBD lithiasis.2) It does not lead to bacterial or “chemical” cholangitis, to “sump” syndrome or to hepatic dysfunction, provided a wide anastomosis is accomplished.3) CDD should only be considered as obsolete after extensive, long-term, prospective, randomized assessments of laparoscopic or combined laparoendoscopic approaches have been shown to be as effective as or superior to CDD.


*HPB Surgery*, 1996, Vol.10, pp.27–33
Reprints available directly from the publisher
Photocopying permitted by license only

© 1996 OPA (Overseas Publishers Association)
Amsterdam B.V. Published in The Netherlands
by Harwood Academic Publishers GmbH
Printed in Malaysia

### Choledochoduodenostomy in the Management of Common Duct Stones or Associated Pathology – An Obsolete Method?

ANTÓNIO CASTRO MENDES DE ALMEIDA, NOEL MEDINA DOS SANTOS and FERNANDO JOSÉ ALDEIA

University Hospital of Santa Maria – Medicina Operatória, Lisbon Medical School, Av.Egas Moniz, 1699 Lisboa Codex Portugal

*(Received 17 December 1994)*

Choledochoduodenostomy (CDD) has been reported as a more effective treatment of CBD stones than T-tube drainage but it is regarded as a last resort or obsolete therapeutic method due to fears of higher mobidity, cholangitis, "sump" syndrome and liver dysfunction. We aimed to assess the aforementioned issues analyzing prospectively our experience from 1976 through Dec.92.

*Methods:* CDD was performed in 89 females and 36 males, aged 60.2±8.7 years, 26 during repeat surgery. Duct stones were the indication in 94, Sphincter of oddi (SO) dysfunction in 23 and obstructive pancreatitis nodule in 8. Peroperative liver biopsies were obtained in 44 patients. The "follow-up" schedule (> 2.5 years in 110) included clinical interview and LFT's on an yearly basis. Ultra sound (USG) was obtained every one or two years. ERC was done in 10 symptomatic patients and in 25 others for protocul purposes. Liver biopsies were taken four to nine years post surgery in 11 patients-five at relaparotomy for non-biliary causes and six percutaneously by fine needle. Ductal mucosa biopsy could safely be performed in one patient 10 years after surgery. The long-term results were classified as *excellent, good, fair* or *poor. Poor* meant the need for further invasive therapy (resurgery or EST).

*Results:* There were two operative deaths (1.6%). The long-term results (123 survivors) were considered *excellent* in 89, *good* in 22, *fair* in 9 and *poor* in three. Three patients died from unrelated causes and eight others ceased the "follow-up" evaluation three to five years post surgery. All of them were considered as having *excellent* or *good* results. A widely patent anastomosis of approximately 20 mms without mucosal inflammatory changes was documented in every patient assessed via ERC. food "debris" was detected within the distal duct of four patients yet it was easily flushed through the stoma. Normal tissue patterns were observed in all long-term liver biopsies. Likewise the ductal mucosa biopsy failed to reveal any acute or chronic inflammatory changes.

*Conclusions:* 1) CDD is ahighly effective short and long-term treatment of CBD lithiasis.2) It does not lead to bacterial or "chemical" cholangitis, to "sump" syndrome or to hepatic dysfunction, provided a wide anastomosis is accomplished.3) CDD should only be considered as obsolete after extensive, long-term, prospective, randomized assessments of laparoscopic or combined laparoendoscopic approaches have been shown to be as effective as or superior to CDD.

KEY WORDS: Common duct lithiasis laparoscopic cholecystectomy bilio-digestive anastomoses cholangitis choledochoduodenostomy

### INTRODUCTION

It is no longer disputed that laparoscopic cholecystectomy is the procedure of choice in a large proportion of patients suffering from symptomatic biliary lithiasis. However controversy still remains as to what should be considered the optimal treatment for patients who in addition to gall-bladder (GB) calculi also harbour common bile duct (CBD) stones. Endoscopic sphincterotomy (EST) became widely accepted as the most appropriate treatment of retained/recurrent duct stones independent of the patient's age and risk. It is also the most judicious treatment for CBD calculi in elderly or otherwise "unfit" patients with GB "in situ".

*Correspondence to*: Dr. A. C. Mendes de Almeida Praça Principe Real, 23-3º Esq, 1200 Lisbon Portugal.

Thus, endoscopic removal of duct stones followed by laparoscopic cholecystectomy is becoming the anticipated scenario of the future because a higher incidence of duct stones occurs in people over 65. Should the duct calculi be detected laparoscopically, the reverse approach seems to be preferable. Nevertheless it has been shown that EST doesn't offer significant advantages with respect to morbidity, mortality and success as compared to conventional CBDE[1], and that "fit" patients should be treated by surgery alone without properative EST [2]. There are two other random and prospective studies that concluded that EST followed by conventional [3] or laparoscopic [4] surgery is not superior to surgery alone. Moreover debate continues to question whether sphincter restenosis and/or stone reformation are a long-term risks following EST [5]. Thus, although EST followed by surgery may be regarded as an appealing approach [6], open CBDE is still preferred by a large number of surgeons, specifically when deciding the most appropriate therapy for a young, "fit", patient for whom the long-term effectiveness of the elected procedure is a major factor.

Among the various surgical modalities at our disposal, choledochoduodenoustomy (CDD) has been shown to be more effective when compared to temporary T-tube decompression in avoiding long-term morbidity secondary to retained /recurrent stones[7-10]. However, many surgeons consider CDD as a last resort and even as obsolete due to fears of increased operative morbidity and mortality risks, long-term bacterial or "chemical" cholangitis and the possible occurrence of the "sump" syndrome. There are also concerns of eventual hepatic dysfunction and/or parenchymatous changes resulting from repeated bouts of cholangitis, from long-standing duodeno-choledochal reflux of enteric secretions, or from the loss of sphincter of oddi activity.

Continuing a previously reported study [11] we have continued to investigate whether or not these fears and/or alegations are justified and to define the short and long-term efficacy of CDD.

### PATIENTS, METHODS

This study reports the analysis of data retrospectively retrieved from January 1973 through December 1975 and prospectively documented from 1976–Dec. 92 according to a protocol. It relates to a consecutive series of 125 CDD which were part of the management of symptomatic biliary lithiasis. Table 1 summarizes the data related to this sub-group of patients. Wgure 1 outlines our approaches to GB and CBD lithiasis during primary and secondary surgery. All of the aforementioned patients were evaluated and operated upon by the same surgical team. The senior author was either the acting surgeon or the first assistant attending the Chief Resident. Thus a uniform set of criteria was ensured.

**Table 1** Summary of data relating to 125 CDD (Jan.73–Dec.92)

| *Data* | *Nr.* | *%* |
|---|---|---|
| Female Patients | 89 | 71.2 |
| Age ($\bar{X}\pm3\times sd$) in yrs | 63.1±3×9.7 | |
| Male Patients | 36 | 28.8 |
| Age ($\bar{X}\pm3\times sd$) in yrs | 58.7±3×13.2 | |
| Nr. of Patients<50 yrs | 28 | 22.4 |
| Nr. of Patients>70 yrs | 32 | 25.6 |
| Primary Surgery | 99 | 79.2 |
| Resurgery | 26 | 20.8 |
| Duct width 10–15 mms | 39 | 31.2 |
| Duct width>15 mms | 86 | 68.8 |
| Operative Morbidity | 12 | 9.6 |
| Operative Mortality | 2 | 1.6 |
| Choledocholithiasis | 94 | 75.0 |
| Obstructive Pancreatitis Nodule | 8 | 6.0 |
| SO dysfunction | 23 | 18.0 |

Other than a "standard" evaluation, the preoperative "work-up included oral cholecystography (OCG) or ultrasonography (USG), serum proteins and biochemistry profiles, and liver function tests (LFT). The clinical suspicion of actual or past presence of intraductal calculi and/or of sphincter of Oddi dysfunction (SO) would prompt us to obtain an intravenous cholangiography (IVC) and/or endoscopic retrograde cholangiography (ERC). We used IVC to confirm or rule out the presence of a dilated CBD (>10–12 mms) as suggested on USG. Most importantly we used it to confirm the possible existence of a sluggish biliary drainage into the gut [11], even though this test is known to be unpredictable in diagnosing duct stones. The criteria for the diagnosis of SO dysfunction and/or an increased likelihood of choledocholithiasis were as follows: 1) complaints of upper abdominal pain after meals, 2) intermittent jaundice (bilirubin >3 mgrms), 3) biochemichal cholestasis, 4) presence, up to 120 minutes, of contrast material within a dilated duct on IVC, 5) indications of a dilated duct on USG. Two or more of these criteria complementing our clinical assessment would prompt us to perform an ERC and/or a thorough intra-operative search for the actual presence of intra-ductal calculi. This evaluation would include a wide kocherization, a careful extra-ductal and pancreatic head palpation, a direct measurement of the duct diameter with a ruler above the

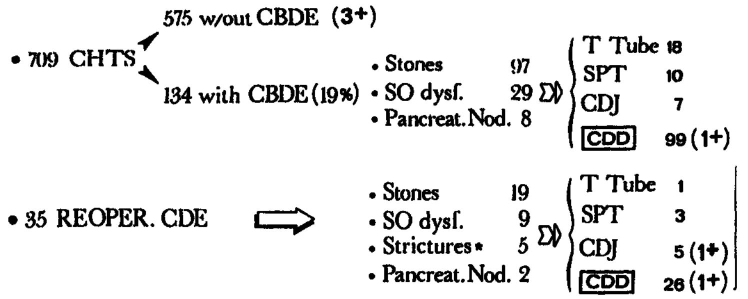

**Figure 1** Schematic outline of the surgical approaches to symptomatic biliary lithiasis during primary and secondary surgery (Jan.73-Dec.92)
CHT-Cholecystectomy; CDE–Common Duct Exploration
SPT–Sphincteroplasty CDJ–Choledochojejunostomy
+–Operative Death; *–Referred from elsewhere

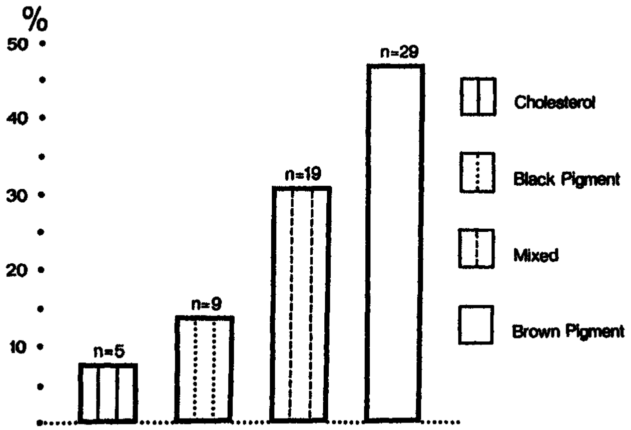

**Figure 2** Percentage of various types of duct calculi according to the morphological criteria of Madden [12], prospectively classiffied (Jan.79–Dec.84)

cystic-choledochal junction and, in selected instances, an operative cholangiogram (OPC). Operative USG or choledochoscopy were no used.

When deemed necessary a longitudinal choledochotomy, following guidelines previously described [11], would confirm or rule out the actual presence of stones and/or inflammatory changes such as thickened duct walls, fibrin strands, hyperemic mucosa or biliary "mud". As reported before [7,10], and herein illustrated in Figure 1 we broadened the indications for CDD in the management of dilated (>10–12 mms) stone-containing ducts. The choledocho-duodenal anastomosis and corresponding technical requisites were already reported [11]. The fundamental aim was the construction of a wide (>20 mms), side-to-side, well blood-supplied anastomosis. Stents were never used. The detection of fibrotic scars or inflammatory changes secondary to duodenal peptic ulcer disease or cholecystocholedocho-duodenal fistula or any other anatomical distortion contraindicated performing a CDD on various occasions. Thus, some other form of biliary drainage was considered (Figure 1).

Peroperative liver biopsies were obtained upon written consent in 44 patients. From January 1979 through December 1984 we prospectively classified, as per Madden's criteria [12], the intra-ductally detected stones. As documented in Figure 2 these were classified as 1) pure cholesterol stones, 2) brown pigment, calcium bilirubinate, stasis stones, 3) black pigment calculi and 4) mixed type of stones. The prospective "follow-up" schedule, covering more than four years in 91 patients and over 12 years in 38 (Fig. 3), included clinical interview by independent observer and LFT's (same as preoperatively) every four to six months for one year and yearly thereafter. USG was obtained every one or two years. ERC was deemed necessary in 10 symptomatic patients and, for protocol study purposes, was performed in 25 other consenting patients three to six years post surgery. Our aim was to look for retained/recurrent stones, mucosal inflammatory

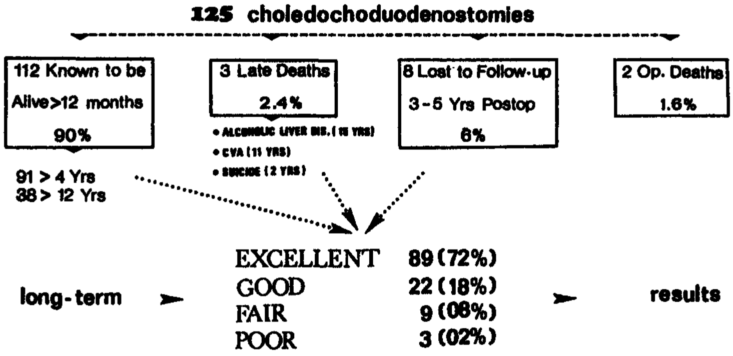

**Figure 3** Length of "follow-up" and classification of long-term results of 123 CDD after a mean number of 9.3±3×4.2 years.

changes at the anastomotic level, the possible presence of food "debris" within the distal "cul-de-sac" or any other anatomical abnormality. Endoscopic biopsy of the duct mucosa could be safely taken in one patient 10 years after surgery. Liver biopsies were obtained four to nine years postoperatively in 11 consenting patients – five at relaparotomy for non-biliary causes: carcinoma of the colon in two, gastric peptic ulcer in one, ventral incisional herina in one and distal esophageal peptic stenosis in one. Six had the biopsy percutaneously by fine needle.

Based on the data, long-term results were classified (Fig. 3). **Excellent** was defined as freedom from any symptoms related to the biliary or upper G.I tract the operation or a complication of the CBDE. **Good** was defined as occasional and minor GI upset, psychosomatic complaints or wound imperfections, with normal LFT,s. **Fair** was defined as significant complaints, manageable by non-invasive treatment, such as those ascribable to the blindsac syndrome or to duodenal-gastric reflux documented on endoscopy, with abnormal LFT's in at least one occasion.Poor was defined as residual or recurrent stones, cholangitis, jaundice and/or severely deranged LFT's requiring further invasive therapy – resurgery or EST.

Operative death or complications were considered as those occurring within 30 days of surgery. Only complications delaying the patient's discharge were accounted for in Tables 1 and 2. A wound infection was defined as one with purulent discharge irrespective of negative bacteriology. Acute cholangitis was diagnosed when the corresponding clinical syndrome (jaundice, high spiking fever with rigors, sepsis, leukocytosis) was confirmed by deranged LFT's and USG. The persistence of bile drainage through the operative wound or out of a drain site that lasted for more than five to six days or its radiographic documentation by contrast extravasation, was taken as evidence of

**Table 2** Postoperative Complications in 123 Patients (two operative deaths not included)

| *Complication* | *Nr.* | % |
|---|---|---|
| *Wound Infection | 5 | 4.1 |
| *Pneumonia | 2 | 1.6 |
| *Congestive Heart Failure | 2 | 1.6 |
| *Biliary Fistula | 2 | 1.6 |
| *Sepsis | 1 | 0.8 |
| *Total | 12 | 9.8 |

biliary fistula. The causes of death were established at postmortem examination.

### SHORT TERM RESULTS

There were two operative deaths (1.6%). A woman of 74 died on the seventh postop day of primary surgery from massive upper GI bleeding. An intact stoma was documented during autopsy. A woman of 62 died on the third postop day after resurgery. Necrotizing pancreatitis with anastomotic disruption was observed on postmortem examination. This patient had had several episodes of pancreatitis and jaundice after having been submitted to cholecystectomy, choledocholithotomy and T-tube decompression at another hospital three years prior to being admitted to our Institution with jaundice. Of the remaining 123 patients, 12 (9.8%) developed significant morbidity (Table 2).

In 44 patients clinical and biochemical evidence of jaundice and cholestasis was discernible on the day of admission. Acute, non-suppurative, cholangitis was the presenting clinical syndrome of 14 patients (11.2%). Although under antibiotic coverage, this syndrome completely subsided only three to five days after CDD had been performed in five of the aforementioned patients.

Twenty-nine of 62 choledochal stones (46.7%) were considered to be of pure brown pigment type.

Evidence of peripheral layers of calcium bilirubinate deposited around a central hard nucleus was observable in 19 others (30.6%), as illustrated in Figure 2.

Evidence of cholestasis, suggesting large duct obstruction, was detectable in all 44 peroperative liver biopsies.

### LONG-TERM RESULTS

Three patients were considered to have had a **poor** result while most remained asymptomatic (72%) one to 19 years (9.3±3×4.2) after having undergone a CDD (Fig. 3).

A widely patent, oval or round-shaped anastomosis was documented in all 35 patients undergoing long-term endoscopic assessment. Endoscopically discernible inflammatory changes, both distant and adjacent to the anastomotic junction, were not observed on the duodenal or ductal epithelial linings. A ductal mucosa biopsy 10 years after CDD revealed a normal histological pattern. Specifically, hyperplasia and hypertrophy of the mucous glands, which have been described as striking features of chronic choledochitis [13], were noticeably absent.

Although easily floating in and out the stoma, food "debris" could be documented in four patients who were otherwise asymptomatic. Retained/recurrent stones could not be detected on long-term ERC. Three patients in whom we knowingly left behind distally impacted calculi deemed to be irretrievable at the time of surgery also were found without stones on ERC examination.

Normal tissue patterns were seen in all specimens of 11 patients evaluated by liver biopsy more than four years after surgery although evidence of cholestasis suggesting large duct obstruction had been documented on peroperative liver biopsy in all of them.

Out of 123 long-term survivors, three patients showed persistent evidence of biochemical and clinical cholestasis requiring further invasive therapy. All of the other patients maintained normal LFT's (Table 3) one to 19 years (9.3±3×4.2 sd) post CDD.

### DISCUSSION

When treating a patient suffering from symptomatic CBD stones, one of several approaches may be considered. Classically, a laparotomy, cholecystectomy and CBDE followed by one of various forms of temporary or definitive drainage of the biliary tree is performed. Another type of management is an EST sometimes followed by laparoscopic or conventional cholecystectomy[14]. Laparoscopic choledochotomy and/or transcystic clearance of duct stones is yet another possible approach [15] though it needs further clinical assessment. If the surgeon is treating an elderly or other higher risk patient the favoured option is the endoscopic approach. This option is also preferred in the presence of retained/recurrent calculi, independent of the patient's age and risk. A quite different challenge is raised when treating a young, "fit" patient in whom a more aggressive stone forming diathesis can be expected thus increasing the possibility of acute cholecystitis or of long-term reformation of intraductal stones. In this particular situation, an open CBDE is envisaged in many centers as the most reliable treatment modality. As we have already reported [7, 10] we favor a liberal utilization of a definitive decompression of the biliary tree after choledocholithotomy, preferably by way of a CDD, when treating such a patient. EST and CDD are techniques that overcome the problems created by the malfunctioning SO, specifically the retention of missed stones or of their reformation as a result of retrograde sphincter pressure waves [16] and/or bile stasis and repeated bouts of infection [7, 10, 17]. Cetta's study [17] is particularly illustrative of this outcome. Our findings (Fig. 2) fully corroborate those of Cetta.

**Table 3** Mean values of long-term results of LFTs in 123 patients submitted to CDD

| *LFT* | *Normal range* | *Values observed in 120 patients ($\bar{X}$±3×sd)* | *Values observed three "outliners" +* |
|---|---|---|---|
| *Bilirubin 1' | 0–4 μmol/l | 2.2±3×1.6 | 16 ; 18 ; 22 |
| * Bilirubin 30' | 2–20μmol/l | 11.9±3×6.2 | 62; 68; 78 |
| * Alk.Phosphat. | 30–90 IU/l | 65.3±3×17.5 | 320; 340; 360 |
| * GGT | 5–38IU/l | 26.3±3×10.0 | 120; 180; 198 |

GGT – Gamaglutamiltranspeptidase
+ Three patients classified as **poor** results
– the only ones out of the range of normalcy

It is undisputable that EST is more comfortable and offers a shorter hospitalization than a formal operation. However the short-term effectiveness of retrograde EST has recently been shown [18] to be less than perfect when compared to that of the current study. Although all-comers were included in ours as well as in the Manchester series, the immediate morbidity and mortality rates were not significantly different. However, a mean number of 1.9 EST session were needed to achieve a less than optimal 81.6% rate of complete ductal stones clearance. This data compares unfavorably to what we and others have accomplished with CDD [19, 20]. The complication and mortality risks of EST have been variously defined and are therefore not easily interpreted. The mortality of this procedure has been reported [18, 21] as occurring at a rate around 2% when the same criteria utilized in surgical series is adopted. It's worth emphasizing that in one of these series [21], which included all-comers, the mean age of the patients was within the same range as in the current series. Possibly of greater significance is the fact that the long-term effects of EST remain as yet incompletely clarified [5], the rate of restenosis and thus the increased chances of stone reformation having been described as ranging from 3 to 10% [22,23]. It must be stressed that two recent random and prospective studies [3, 4] concluded that the laparoendoscopic appraoch is not superior to a standard cholecystectomy and CBDE, even in terms of short-term efficacy. Another study [24] showed that the stone reformation is, actually, enhanced after EST and suggested that a non-functioning, partially restenosed, SO was responsible. The data in our study does not support the contention that bilio-duodenal bypass leads to hepatic dysfunction resulting from repeated bouts of cholangitis, nor does it support the claim that significant gastrointestinal complaints such as persistent diarrhoea with nutritional impairment occur. These complications, which have been described as the sump or blindsac syndrome, presumably derive from stasis and refluxed duodenal contents in to the terminal common duct, with bacterial overgrowth enhancing bile salt deconjugation. This phenomenon leads not only to diarrhoea, as observed in the "blind-loop" syndrome arising anywhere else in the gut, but it also facilitates deposition and reformation of calcium bilirubinate stones [17, 24]. In the present series, though, only three patients were considered as **poor** results consequent to repeated bouts of cholangitis. Recurrent stones could not be documented in spite of an exhaustive search. A very wide anastomosis could possibly be the explanation.

In one of the patients considered to have had a **poor** result, severely disturbed LFT's antedated our first surgical procedure. At the time, a left hepatic duct stenosis, presumably deriving from previous surgery, was missed. We eventually corrected this patient's cholangitic syndrome with a hilar Roux-Y hepaticojejunostomy followed by long-term good results. A poorly draining CDD, sited too high in the common hepatic duct was the reason for a **poor** result in one other patient. Long-term good results also ensued after a Roux-Y hepaticojejunostomy in this patient. Up to this time we are unable to define the exact cause for the third poor result. Repeated ERC examinations failed to disclose any evidence of stoma stenosis, recurrent stones or any other form of biliary obstruction. The long-term results after EST were not entirely satisfactory in this patient.

The blind-sac syndrome can be avoided by a wide anastomosis. This prevents stasis, avoids the build-up of excessive intra-ductal pressure and permits a free flow of common duct contents including duodenal refluxate as well as eventual retained/recurrent calculi back into the duodenum. Using the triangular technique, as previously described [11], it is technically feasible to construct a biliary-duodenal anastomosis of sufficient diameter as to prevent these complications even on a duct 10 to 12 mms wide. The possible long-term effects on liver function allegedly resulting from loss of the odditic sphincter activity remain to be determined. Certainly there was no evidence in our study.

In conclusion our data, corroborating others [8,9,19,20], indicate that CDD is a very safe procedure with morbidity and mortality risks similar to those of EST whenever similar cohorts of patients are compared. The alleged late complications of liver dysfunction and blind-sac syndrome are low enough in incidence to warrant a broadening of indications for CDD in young patients. It's beyond any dispute that EST followed by laparoscopic or conventional cholecystectomy is the most judicious treatment for a great number of patients with symptomatic duct lithiasis. It's worth emphasizing that most patients with this clinical syndrome belong to an age group where other fatal non-biliary diseases are likely to occur before So restenosis and/or stone reformation do so. The declining number of CDD in the current series, as illustrated in figure 4, clearly directs our thoughts to this respect. However in a young, "fit", patient it is our contention that a carefully performed CDD is the most appropriate treatment of most dilated stone-containing ducts. With the current emphasis on laparoscopic procedures

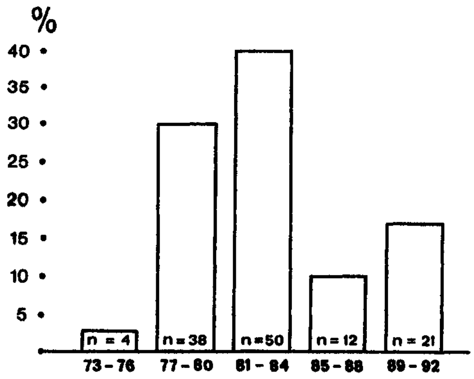

**Figure 4** Number and percentage of CDD performed along five sequential periods of four years each.

and the rapidly advancing technology it is our firm belief that we will be able to perform a correct CDD entirely by laparoscopic means in the near future. Until then, though, only after long-term, extensive, prospective assessments of any other form of treatment for duct lithiasis in a young population, be it conventional, purely laparoscopic or combined laparoendoscopic, are able to show an efficacy similar or superior to that herein reported with conventional CDD can this procedure be considered as obsolete.

### REFERENCES

1. Miller B.M, Kozarek R.A, Ryan Jr J.A, Ball T.J and Traverso LW (1988), Surgical versus Endoscopic Management of Common Bile Duct Stones. *Ann. Surg,* **207**: 135–141.
2. Neoptolemos J.P, Shaw D.E and Carr-Locke D.L (1989), A Multivariate Analysis of Preoperative Risks in Patients with Common Bile Duct Stones. *Ann. Surg,* **209**: 157–161.
3. Stain S.C, Cohen H, Tsuishoysha M, Donovan A.J (1991), Choledocholithiasis: Endoscopic Sphincterotomy or Common Bile Duct Exploration. *Ann. Surg,* **213**: 627–634.
4. Stiegman G.V, Goff J.S, Mansour A, Pearlman N, Reveille R, Norton L (1992), Precholecystectomy Endoscopic Cholangiography and Stone Removal is not superior to Cholecystectomy, Cholangiography and Common Duct Exploration. *Am. J. Surg,* **163**: 227–230.
5. McEntee G, Grace Pa, Bouchier-Haynes D. (1991) Laparoscopic Cholecystectomy and the Common Bile Duct (Leading Article, Royal College of Surgeons of Ireland). *Br. J. Surg,* **78**: 385–386.
6. Heinerman P.M, Boeckl O, Pimple W. (1989) Selective ERCP and Preoperative Stone Removal in Bile Duct Surgery. *Ann. Surg,* **209**: 267–272.
7. Almeida A.M, Gracias C.W and Santos N.M (1982); Definitive Decompression of the Biliary Tree-Preferred Approach to Management to Choledocholithiasis Am. *J. Gastroenterol,* **77**: 941–946.
8. Crumplin W.G, Jenkinson L.R, Kassab J.Y, Whitaker C.M, and Boutiahi F.H. (1985) Management of Gallstones in a District General Hospital. *Br. J. Surg,* **72**: 428–432.
9 Sheridan W.G, Williams H.O, and Lewis M.H. (1987) Morbidity and Mortality of Common Bile Duct Exploration. *Br. J. Surg,* **74**: 1095–1099.
10. Almeida A.M, Aldeia F.J, Santos N.M. and Gracias C.W. (1992) Standard Surgical Approaches to Primary Choledocholithiasis-Definitive versus Temporary Decompression. *HPB Surgery,* **6**: 35–49.
11. Almeida A.M, Cruz A.G, Aldeia F.J. (1984) Side-to-Side Choledochoduodenostomy in the Management of Choledocholithiasis and Associated Pathology-Facts and Fiction, *Am. J. Surg,* **147**: 253–258.
12. Madden J.L. (1973) Common Duct Stones. Their Origin and Surgical Management, Surg. Cl. *North Amer,* **53**: 1095–1113.
13. Schein C.J, Mahadevia P.A. (1979) Surgical Significance of Histopathology of the Common Bile Duct, *Am. J. Surg,* **137**: 763–767.
14. Martin D.F, Tweedle D.E.F. (1987) Endoscopic Management of Common Duct Stones without Cholecystectomy, *Br. J. Surg,* **74**: 209–211.
15. Phillips E.H, Carroll B.J, Pearlstein A.R, Daykhovsky L. and Fallas M.J. (1993) Laparoscopic Choledochoscopy and Extraction of Common Bile Duct Stones, *World J. Surg,* **17**: 22–28.
16. Tooulli J, Geenen J.E, Hogan W.J. (1982) Sphincter of Oddi Motor Activity: A comparison between Patients with Common Bile Duct Stones and Controls, *Gastroenterology,* **82**: 111–117.
17. Cetta F. (1991) The Role of Bacteria in Pigment Gallstone Disease, *Ann. Surg* **213**: 315–326.
18. Lambert M.E, Betts C.D, Hill J, Faragher E.B, Martin D.F. and Tweedle D.E.F. (1991) Endoscopic Sphincterotomy: The Whole Truth, *Br. J. Surg,* **78**: 473–476.
19. Parrilla, P, Ramirez P, Sanchez-Bueno F *et al.* (1991), Long-Term Results of Choledochoduodenostomy in the Treatment of Choledocholithiasis: Assessment of 225 Cases, *Br. J. Surg,* **78**: 470–474.
20. Escudero-Fabre A., Escallon J.R.A, Sack J, Halpern N.B, and Alderte J.S. (1991) Choledochoduodenostomy-Analysis of 71 Cases followed for 5 to 15 years, *Ann. Surg,* **213**: 635–642.
21. Sherman S, Ruffolo T.A., Hawes R.H. and Lehman G.A. Complications of Endoscopic Sphincterotomy: A Prospective Series with Emphasis on the Increased Risk Associated with Sphincter of Oddi Dysfunction and Non-Dilated Bile Ducts, *Gastroenterology,* 1991; **101**: 1068–1075.
22. Rieman J.F., Lux G., Forster P., Altendorf A., Long-Term Results after Endoscopic Papillotomy. *Endoscopy,* 1983; **15**: 165–168.
23. Cotton P.B. (1984) Endoscopic Management of Bile Duct Stones (Apples and Oranges), *Gut,* **25**: 587–597.
24. Cetta F. (1993) Do Surgical and Endoscopic Sphincterotomy Prevent or Facilitate Recurrent Common Duct Stones Reformation?, *Arch. Surg,* **128**: 329–335.

